# The Question of Linked-Up Illnesses

**Published:** 1914-06-20

**Authors:** 


### NATIONAL INSURANCE ACT DEVELOPMENTS.

### The Question of Linked-up Illnesses.

A circular has recently been issued by the Insurance Commissioners giving instructions to approved societies, and through them to the insured persons, as to the circumstances under which separate attacks of sickness are to be regarded as one continuous illness for the purpose of the payment of sickness benefit. Since a good deal of confusion appears to exist on the subject, and since also a number of subsidiary questions of considerable importance arise out of it, we propose to devote our space this week to a consideration of the whole matter.

Section 8 (5) of the Act of 1911 as amended by Section 12 of the Act of 1913 lays down a rule regarding the payment of sickness and disablement benefit which may be expressed as follows: "When a member of an approved society or a deposit contributor has received sickness benefit or disablement benefit for one illness and falls ill again within twelve months of his recovery, his second illness must be treated as a continuation of the first." In other words the two or more illnesses are to be "linked up" and benefit is to be paid in respect of the period during which the member is incapable of work, without regard to the fact that a short period of recovery has separated two attacks of sickness.

### Why the Rule was Instituted.

Before considering the general effect of this rule and before noticing its application to particular cases, it would be well to consider briefly why such a condition should have been made to govern the payment of sickness and disablement benefit, especially as the condition appears to cause a certain amount of hardship to insured persons. The rule at any rate has the merit of having been justified by long experience, since, except for differences in the length of the minimum period that must elapse between two illnesses, some such rule may be found in practically the whole of the existing Friendly Societies and was in force long before the passing of the National Insurance Act. The main reason for the creation of the rule was that the funds of the societies might be protected from fraud on the part of unscrupulous sick members; the possibility of fraud arises from the fact that it is usual, as in the case of State insurance, to pay a higher rate of benefit during the earlier stages of an illness than during the later. It can easily be seen, for instance, that if no protective measures were adopted an insured person under the Insurance Act, having exhausted his right to sickness benefit, would feel very much tempted to declare off the funds for a short period instead of taking the smaller disablement benefit, and then to declare on again and recommence benefit at the full rate.

The institution of a minimum period of twelve months between two illnesses, though possibly causing hardship in particular cases, effectively puts a stop to this type of fraud by removing all incentive to commit it, and is, therefore, in the highest degree justifiable.

The general effect of the operation of the rule may perhaps best be seen if we quote an illustration given by the Commissioners in their circular before referred to.

### A Case by Way of Example.

"John Smith falls ill on September 1, 1914, and claims sickness benefit. It is found that he had previously received sickness benefit for eight weeks in January and February, 1914. He is, therefore, entitled to sickness benefit *from the first day*, September 1, 1914, for a period not exceeding eighteen weeks. If at the end of those eighteen weeks he is still incapable of work and has been insured for 104 weeks and paid 104 contributions disablement benefit then becomes payable." There are one or two points in connection with this illustration and with the application of the rule to which we would draw particular attention. In the first place it should be noted that in the illustration given, sickness benefit in the case of the second illness is payable from the first day of sickness and not from the fourth day as on a *first* attack; this, of course, naturally follows from the fact that the illness is assumed to have commenced in January, and for the purpose of deciding on the rate of benefit is further assumed to have been unbroken.

### A Point for Insured Persons.

Insured persons should see to it that they receive what is due to them in this respect and that approved societies are not allowed to reap a double advantage. It should furthermore be observed that the period of twelve months is reckoned from the day on which the member recovers from his illness, and not from the date on which he ceased to receive sickness benefit for that illness. This is an extremely important point and is of especial importance at the present moment, since no insured person is yet entitled to disablement benefit. Suppose, for instance, that a member of an approved society fell ill for the first time on February 1, 1913, and continued ill until February 1, 1914, a period in all of some fifty-two weeks. For the first twenty-six weeks of his illness he would receive sickness benefit, but during the remainder of his illness he would receive no weekly payment whatever, since he is not yet entitled to disablement benefit. Then should he again fall ill at any time within twelve months of February 1, 1914, his second illness would not be regarded as a fresh one but as a continuation of the old one; and unless, therefore, in the meantime he had become entitled to disablement benefit he would still receive no benefit, and this, in spite of the fact that considerably over twelve months may have elapsed since he last received any weekly payment at all.

### Qualification for Disablement Benefit.

This leads us naturally to consider the position of such a member with regard to qualification for disablement benefit. No insured person can receive disablement benefit until he has been insured for 104 weeks and has paid 104 contributions. No person can therefore possibly receive disablement benefit until July 1914, and then only if he entered into insurance in July 1912 and has paid a contribution in respect of every week in the intervening period.

For an insured person who joined in July 1912, and who is neither ill nor unemployed until July 1914, these conditions are comparatively simple, but not so in any other case. If, for instance, an insured person falls into arrears through unemployment, the date on which he will first become entitled to disablement benefit will be deferred unless he makes up these arrears. In this connection it should be noted that these arrears may now be made good by payment of the contribution due from the insured person only (i.e., 4d. or 3d. per week, as the case may be), and the employer's contribution is excused. If, however, an insured person is out of employment through being incapable of work he is not considered as incurring arrears, and if, therefore, such a person desires to be entitled to disablement benefit at the earliest possible date he must pay contributions *at the full rate* (that is, the employer's contribution as well as his own) for the whole of the period during which he was incapable of work, irrespective of whether or not during the whole of this time he received benefit. In the hypothetical case just considered, therefore, if the insured person desires to become qualified to receive disablement benefit at the expiration of 104 weeks from the date of his entry into insurance, he must pay 52 contributions of 7d. per week in respect of the whole of the period from February 1913 to February 1914 during which he was incapable of work. If he had been merely unemployed during this period the corresponding cost would have been little over one-half this amount. Although this may seem to be a somewhat anomalous state of affairs, yet there is a certain amount of justification for the position, inasmuch as a person incapable of work produces a far greater strain on the funds, having already received a considerable sum in sickness benefit, and is likely to produce a still greater strain in the future, especially if the illness becomes chronic.

### Sickness Benefit not Actually Received.

There are three cases in which a member may not have actually received sickness benefit for an illness, but will be considered as having received such benefit for the purpose of deciding whether or not an illness is to be linked up. These are:—

(1) Where during his illness the member has been in an institution such as a hospital, infirmary, etc., and the amount of the benefit which would ordinarily have been payable to him has been applied in some other way, such as in payment to his dependants or to the institution under an agreement.

(2) In cases where employers have made special arrangements to themselves pay full wages or two-thirds wages during illness and a corresponding reduction has been allowed in the contribution. In these cases the member is not entitled to sickness benefit from his society until the employer's liability to pay full remuneration (or two-thirds remuneration as the case may be) has been discharged, but he is to be considered as having received sickness benefit for six weeks before the date on which he becomes entitled to benefit from the society.

(3) Where, in the case of a member employed in the Mercantile Marine, there has been a period of illness during which the member was entitled to medical attendance and medicine as well as maintenance at the cost of the owner of his ship.

The equity of this is hardly disputable.

### Compensation Cases.

One other aspect of the subject remains to be considered. When an insured person receives compensation from his employer in respect of any illness, approved societies are only liable to pay such an amount each week as will, together with the compensation received, amount to the weekly benefit that the insured person would ordinarily have been entitled to receive under the Insurance Act. Thus, if the insured person would in the ordinary way have been entitled to 10s. per week sickness benefit, and receives in respect of a certain illness 7s. 6d. per week compensation from his employer, the approved society to which he belongs would only be called upon to pay 2s. 6d. per week for twenty-six weeks in respect of that illness. Now the Act of 1913 lays down with respect to a person such as this that if he recover from his illness before having received twenty-six weeks' benefit, and again falls ill within twelve months of his recovery, he is to be treated as having received sickness benefit, not for the full period for which benefit was paid at the reduced rate, but for a reduced period proportionate to the reduced sickness benefit received. Thus in the illustration just given, if the insured person recovered from the illness for which he received compensation after the lapse of eight weeks, but fell ill again within the ensuing twelve months, he would not for the purpose of linking up the illnesses be considered as having received eight weeks' sickness benefit, but only two weeks' benefit, since the total amount received during the eight weeks from the society was only £1. Sickness benefit would thus be paid in respect of the second illness at the ordinary rate from the first day of incapacity, and would continue (so long as the member remained ill) until the total amount paid, including the first illness, was equal to twenty-six weeks' benefit at the ordinary rate.

It should also be noted that when the weekly value of the compensation received is equal to or greater than the sum payable as sickness benefit, the insured person does not come on the funds of his society, and the period of incapacity covered by such compensation is not to be reckoned in calculating the right to any future sickness benefit.

